# Do executive functions predict physical activity behavior? A meta-analysis

**DOI:** 10.1186/s40359-023-01067-9

**Published:** 2023-02-02

**Authors:** Ceren Gürdere, Tilo Strobach, Massimiliano Pastore, Ines Pfeffer

**Affiliations:** 1 Department of Psychology, Bilkent University, Ankara, Turkey; 2 Institute of Cognitive and Affective Neuroscience (ICAN), Medical School Hamburg, Am Kaiserkai 1, 20457 Hamburg, Germany; 3 Department of Developmental Psychology and Socialisation, University of Padova, Padova, Italy

**Keywords:** Cognitive functions, Inhibition, Updating, Shifting, Exercise, Systematic review, Self-regulation

## Abstract

**Background:**

Executive functions (EFs) are important determinants of health behaviors. In the present study, a meta-analysis was conducted to investigate the relationship between EFs and physical activity (PA) behavior.

**Methods:**

Systematic searches were carried out in PsycInfo, MEDLINE, and SPORTDiscus databases throughout April 2021. Prospective empirical studies conducted with general healthy populations across the lifespan, which reported the relationship between baseline EFs and later PA behavior were selected.

**Results:**

Eight studies were found eligible. Results of the multilevel meta-analysis revealed a small but significant total effect size for EFs on PA behavior of *z* = 0.12. High heterogeneity was observed among studies. When potential moderators were tested, residual heterogeneity remained significant and the effects of the moderators were not significant. The effect size dropped when accounted for publication bias.

**Conclusions:**

Despite limitations, the study provided evidence for EFs’ determinant role on PA behavior. More research is however encouraged to inform PA promotion programs that are well-prepared for individual differences in EFs.

**Supplementary Information:**

The online version contains supplementary material available at 10.1186/s40359-023-01067-9.

## Introduction

Physical activity (PA) is one of the most important health behaviors for the prevention and the therapy of widespread non-communicable chronic diseases such as coronary heart disease, stroke, diabetes mellitus, back pain, and mental disorders [1, 2]. PA is defined as any bodily movement that is produced by skeletal muscles and significantly increases the basal metabolic rate [3]. Despite extensive evidence documenting the positive effects of PA on physical and mental health, too many people in western industrialized countries are physically inactive (e.g., adults not reaching at least 150–300 min of moderate-intensity aerobic physical activity a week) [4–7]. This insufficient activity status emphasizes the importance for sound knowledge about relevant factors that might facilitate or hinder PA behavior, as these factors should inform the development of effective PA promotion programs. In the context of dual-process models and their implicit and explicit processes, researchers however became increasingly interested in the role of executive functions (EFs) as a determinant of PA behavior only rather recently [8, 9].

### Executive functions and the self-regulation of health behavior

EFs are a set of general-purpose control mechanisms that regulate the dynamics of human cognition and action. These functions are important to study because they are a core component of self-control or self-regulation ability, which has been shown to have broad and significant implications for everyday life. Particularly, individual differences in these processes may predict health behaviors and the translation of intentions into action, such as PA [10, 11]. Additionally, neuroscience studies showed that grey matter volume and activation in the lateral prefrontal cortex (lPFC) are linked with EF performances [12]. In detail, it was documented that larger lPFC volume and activation in lPFC regions predicted higher adherence to PA [13].

In their unity/diversity framework, the complexity of different situations and processes involving the EF construct were systemized primarily in three domains: inhibition, updating, and shifting [14–17]. *Inhibition* is related to deliberate overriding of dominant or prepotent responses, *updating* refers to monitoring and manipulating working memory contents, and *shifting* is associated with switching flexibly between different tasks or mental sets (i.e., cognitive flexibility). The unity/diversity framework states that although the executive domains tap some common variability (i.e., the unity component), they also show separability (i.e., the diversity component). This common variability and separability are assessed by analyzing behavioral performance in EF tests.

It is assumed that EFs support the self-regulation of goal-directed behavior in process-oriented terms by organizing information and behavior to effortfully overcome short-term gratifications not in line with the attainment of long-term goals. Self-regulation entails 1) a standard or a goal that individuals endorse, mentally represent, and monitor, 2) sufficient motivation to invest effort into reducing discrepancies between standards and actual states, and 3) sufficient capacity to achieve the goal or the standard by reducing the discrepancy despite temptations and barriers that might arise [18].

In detail, one main aspect of successful self-regulation is the ability to actively inhibit or override behavioral responses (such as [unhealthy] habits and impulses) that are incompatible with one’s (healthy) goals [11]. In experimental lab contexts, this inhibition component was assessed by the go/no-go task [19] and the Stroop task [20], among others. The go/no-go task requires the inhibition of responses on one set of stimuli while there are speeded responses on other stimuli. Alternatively, in the Stroop task, participants are instructed to respond to the ink of color words; these color words are congruent (e.g., GREEN in green ink) or incongruent (e.g., GREEN in red ink). Typically, reaction times in incongruent trials are larger than in congruent trials (i.e., the Stroop effect), indicating the requirement to inhibit or to override the tendency to produce a more dominant or automatic response on naming the color word in this task. Studies have shown that participants with low levels of inhibition in these experimental inhibition tasks (i.e., large Stroop effects in the Stroop task or high RTs in the go/no-go tasks) are less successful at adhering to regular exercise classes [21] or at translating their PA intentions into action [22, 23].

However, in behavior beyond PA, several other researchers have struggled to replicate the finding of an association between inhibition and behavior or the intention–behavior gap [22, 24, 25], i.e. the association between inhibition and the difficulties to translate PA intentions into action, respectively. Furthermore, it is discussed that behavioral inhibition might play a less important role in positive health behaviors compared with the role in negative or risky behaviors, whereas updating might be more important for the initiation of positive health behaviors compared to negative health behaviors [24, 26]. It is suggested that higher updating ability is associated with a smaller PA intention-behavior gap [26]. Through the updating function, mental representations of positive health behavior goals and means of goal achievement can be kept active and available for systematic processing [11]. Furthermore, updating might facilitate regulation of affect that is incongruent with goal achievement. On the other hand, the shifting function might benefit self-regulation through flexibly adapting behavior in response to changing circumstances, instead of trying to follow rigid plans or means for goal attainment and by seizing new opportunities as they arise. Although there is an integration of inhibition with the domains updating and shifting in the unity/diversity framework [16], there are investigations needed that associate all of these domains with PA behavior and the intention-behavior gap in a systematic and elaborative way.

### Recent meta-analyses on executive functions and physical activity behavior

Several meta-analyses provided evidence for a substantial relationship between PA behavior and EFs [27–31]. However, these meta-analyses mainly focused on the question, whether acute bouts or regular PA can improve cognitive functions (such as EFs). To date, much fewer studies and reviews scrutinized the question, if EFs might also support the execution of PA via self-regulatory processes [8]. Even though there is convincing theoretical overlap between EFs and self-regulatory processes, empirical evidence for this direct relationship between EFs and PA behavior or the moderating effect of EFs on the intention-behavior relationship is still scarce.

The meta-analysis of Gray-Burrows et al. [32] was the first that synthesized existing studies that examined this relationship in healthy adults for different health behaviors, differentiating between health-protective (e.g., fruit/vegetable consumption, PA, sleep) and health-damaging (e.g., addictive behaviors, alcohol consumption, smoking, snack consumption) behaviors. Six studies examining PA behavior were included. The overall effect size for the association between EFs and PA behavior was calculated at *r* = 0.085. However, this meta-analysis mixed both cross-sectional and prospective studies. This mix is critical, since particularly cross-sectional studies are the least suitable to make causal statements about the direction of the relationship, as they cannot address the temporal relationship between the predictor (e.g., EFs) and a behavior (e.g., PA behavior). Knowledge about the causal relationship is however required to inform about the development of effective PA promotion programs to increase this activity and to reach and maintain health benefits. Thus, an elaborate analysis of the causal relation between EFs and PA from a general perspective of a meta-analysis is lacking.

### The present study

The current meta-analysis aimed to examine the EFs as predictor of PA behavior and to examine possible moderators of this relationship. The present study was based on the well-established model of EFs [15–17] in which the complexity of different situations and processes involving the EF construct was systematized in three unified but diverse domains, namely inhibition, updating, and shifting.

## Methods

This meta-analysis was conducted in accordance with the Preferred Reporting Items for Systematic Review and Meta-Analysis 2020 Statement [33].

### Search strategy

Systematic searches were conducted throughout April 2021 with *physical activity, exercise, sport, and executive functions* as keywords combined by Boolean operators as follows: (physical activity OR exercise OR sport) AND executive function* in PsycInfo, MEDLINE, and SPORTDiscus electronic databases; we focussed on those databases because they are primarily specialised in studies in the relevant field of EFs and PA. Also, forward and backward tracking was carried out.

### Study selection and eligibility

In the first step, the results of the database search were screened for eligible studies by reviewing their titles and abstracts. Then the full texts of these studies were examined according to the following inclusion and exclusion criteria by three independent reviewers. Studies were included if (I) study design was prospective, (II) baseline EF and later PA behavior were measured with direct (e.g., performance tests for EFs, accelerometer data for PA) and/or indirect (e.g., self-report questionnaires) tools, (III) the direct relationship between baseline EF and later PA behavior was reported, (IV) the study was conducted in a healthy population. Studies were excluded if (I) the study design was cross-sectional, (II) the study sample was of special population (e.g., psychological disorder, cognitive impairment), or physical health condition that constitutes medical contradiction to engaging in PA. To address this research question, we refrained from including cross-sectional studies, as these studies do not allow us to draw any conclusions about the direction of the relationship between EFs and behavior. Instead, we focused on prospective study designs where baseline EFs and PA behavior at a later point were assessed, including longitudinal studies, intervention studies, and RCTs as these latter types of studies are most informative about a causal relationship between EFs and PA. Authors were contacted to request the direct correlational relationship result where it was not present.

### Quality assessment of the studies

Each study was assessed with regard to quality with an instrument with 13 items adapted from Favieri et al. [34] and Tooth et al. [35]. The 13 items were rated independently by two experts (0 = poor, 1 = fair, 2 = good, or as ? = not stated/unclear). The items mainly focused on the methodology of the studies (e.g., ‘Do the measures of PA reflect what we want them to (validity)?’) but also on a clearly stated aim as well as on the discussion of the results and the conclusions. Disagreements between the two experts’ ratings were resolved through discussion. An uncertainty index was computed with the sum of not stated/unclear rated items. Furthermore, a relative uncertainty index was calculated and indicated the percentage of uncertain items.

### Statistical analyses

Data analyses were conducted with the Metafor package [36] in R statistical software [37]. For the EF scores where lower scores indicated higher performance (e.g., Stop Signal task), the Pearson correlation coefficients between EF score and PA behavior were reversed to quantify EF performance and PA relationship. Then, a multilevel meta-analysis with effect sizes nested within studies was performed. We examined the heterogeneity across studies with *Q* statistic, where a significant *Q* value indicates significant heterogeneity of results among studies [38]. Next, the magnitude of the heterogeneity was obtained with *I*^2^ index [39].* I*^2^ estimates the proportion of observed variance that reflects differences in effect sizes [40]. High *I*^2^ index value reflects different results across studies due to such as different designs and constructs whereas low *I*^2^ index value points to similar results across studies. *I*^2^ index value below 50% is considered low, 50–75% moderate, above 75% high. We tested for potential moderators using multivariate effects models. Lastly, publication bias was assessed with the “trim and fill” funnel plot method [41, 42].

## Results

**Fig. 1:**
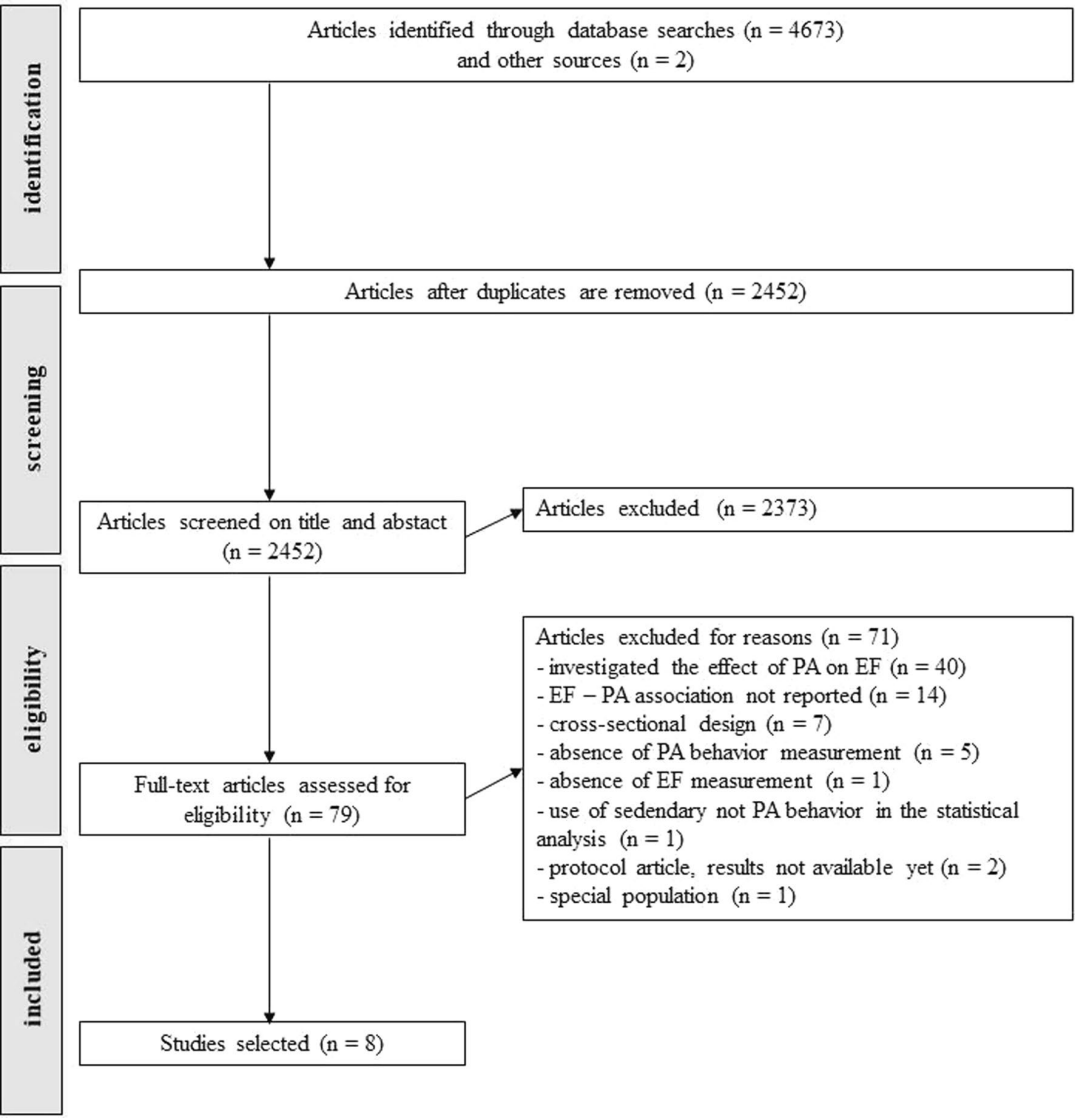
Flow diagram of the study selection process

In the first step, the database search yielded 4673 results and among them, 79 potentially eligible studies. More than half of those 79 studies investigated improvements in EFs with PA (in contrast to our aim of testing the impact of EF on PA) and were thus excluded. The second most common exclusion reason was the absence of reporting direct EF-PA relationships. From among the 79 studies, a total of 8 studies were considered eligible. The flow chart is presented in Fig. 1.

Characteristics of the studies included in the meta-analysis regarding design details, sample size, mean and range of age at baseline, EF and PA measures are presented in Table 1. As noted, most of the studies were observational (1–6), one was RCT (7), and one was intervention (8). Samples consisted of young adults (1, 4, 5, 6, 7), older adults (8), mixed young and older adults (3), and children (2). Sample sizes ranged from 32 to 6069, and the duration of the study from 1 week to 6 years. Almost all of the studies employed performance tests of EFs (2–8), one study used both performance tests and self-report measure (5), and one study used a self-report measure only (1). For PA behavior, half of the studies used self-report measures (1, 4, 6, 7), two recorded accelerometer derived data (2 and 3), one both (5), and one (8) attendance record (see also Additional file 1).

**Table 1:** Summary of study characteristics

Study	Design	Sample	EF measurement	PA measurement
1. Frye and Shapiro [43]	Prospective observational T: 1 week	N: 220 M_age_:19.4 R_age_: 18–25	Barkley Deficits in Executive Functioning Scale	Self-reported PA with International Physical Activity Questionnaire
2. Stautz et al. [44]	Prospective observational T: 6 years	N: 6069 M_age_: 7	Stop signal, counting span, opposite world and sky search tasks	Average daily minutes of MVPA recorded using actigraph monitors
3. Hall et al. [45]	Prospective observational T: 1 week	N: 208 M_age_: 45.2 R_age_: 18–89	Stroop and go/no-go tasks	Average daily PA recorded by accelerometer
4. Hall et al. [23]	Prospective observational T: 1 week	N: 64 M_age_: 19	Go/no-go task	Self-reported number of hours spent in vigorous PA over the past week
5. Loprinzi et al. [46]	Prospective observational T: 1 week	N: 32 M_age_: 21.1 R_age_: 18–45	Tower of London, operation span, stroop, letter-number, and switching tasks	Physical Activity Vital Signs Questionnaire Godin Leisure-Time Questionnaire Accelerometer-derived light PA
6. Pfeffer and Strobach [26]	Prospective observational T: 1 week	N: 118 M_age_: 23.2 R_age_: 18–30	Go/no-go, stop-signal, visual memory, n-back, cueing, and alternating runs tasks	Self-reported number of hours engaged in vigorous PA
7. Pfeffer and Strobach [47]	Prospective RCT T: 1 week	N: 191 M_age_: 22.7 R_age_: 18–34	Go/no-go, stop-signal, visual memory, n-back, cueing, and alternating runs tasks	Self-reported number of hours engaged in vigorous PA
8. McAuley et al. [21]	Prospective intervention T: 12 months	N: 177 M_age_: 66.5 R_age_: 58–81	Dual, stroop, flanker, Wisconsin card-sorting, and switching tasks	Exercise class attendance

Studies are listed in random order. *RCT* Randomized controlled trial, *T* Time frame/duration of the study, *N* Sample size, *M* Mean, *R* Range

The mean agreed quality rating for the eight studies ranged from 1.3 to 1.8 indicating fair to good study quality (Table 2). However, some studies showed substantial uncertainty with regard to study quality (5, 8) as indicated by the (relative) uncertainty index (see Table 2).

**Table 2:**
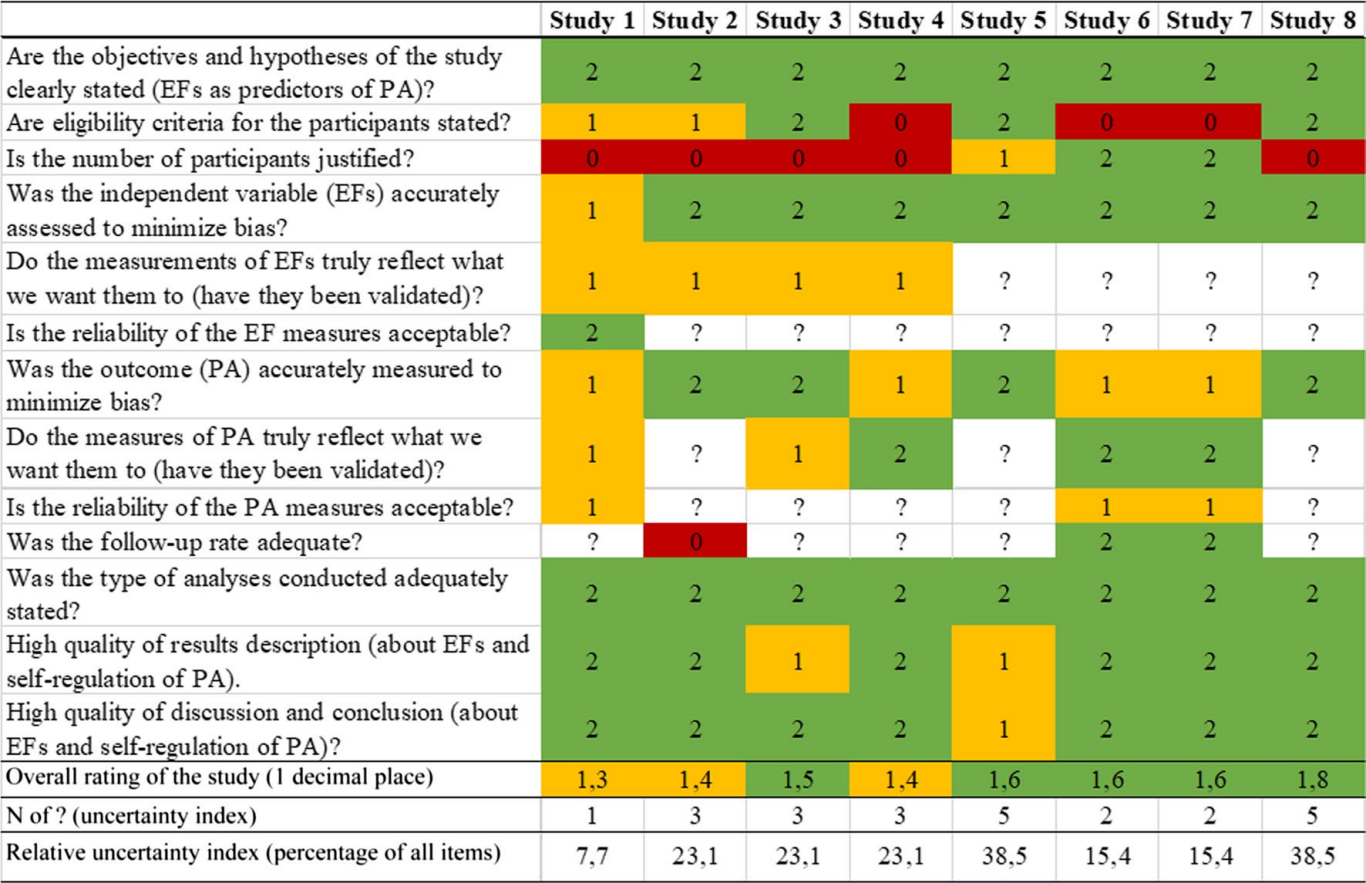
Quality assessment of the included studies

Answering options for each item were Yes (2); Partly (1); No (0); Not stated/unclear (?); Overall rating of the study was assessed as Good (2); Fair (1); Poor (0)

**Fig. 2:**
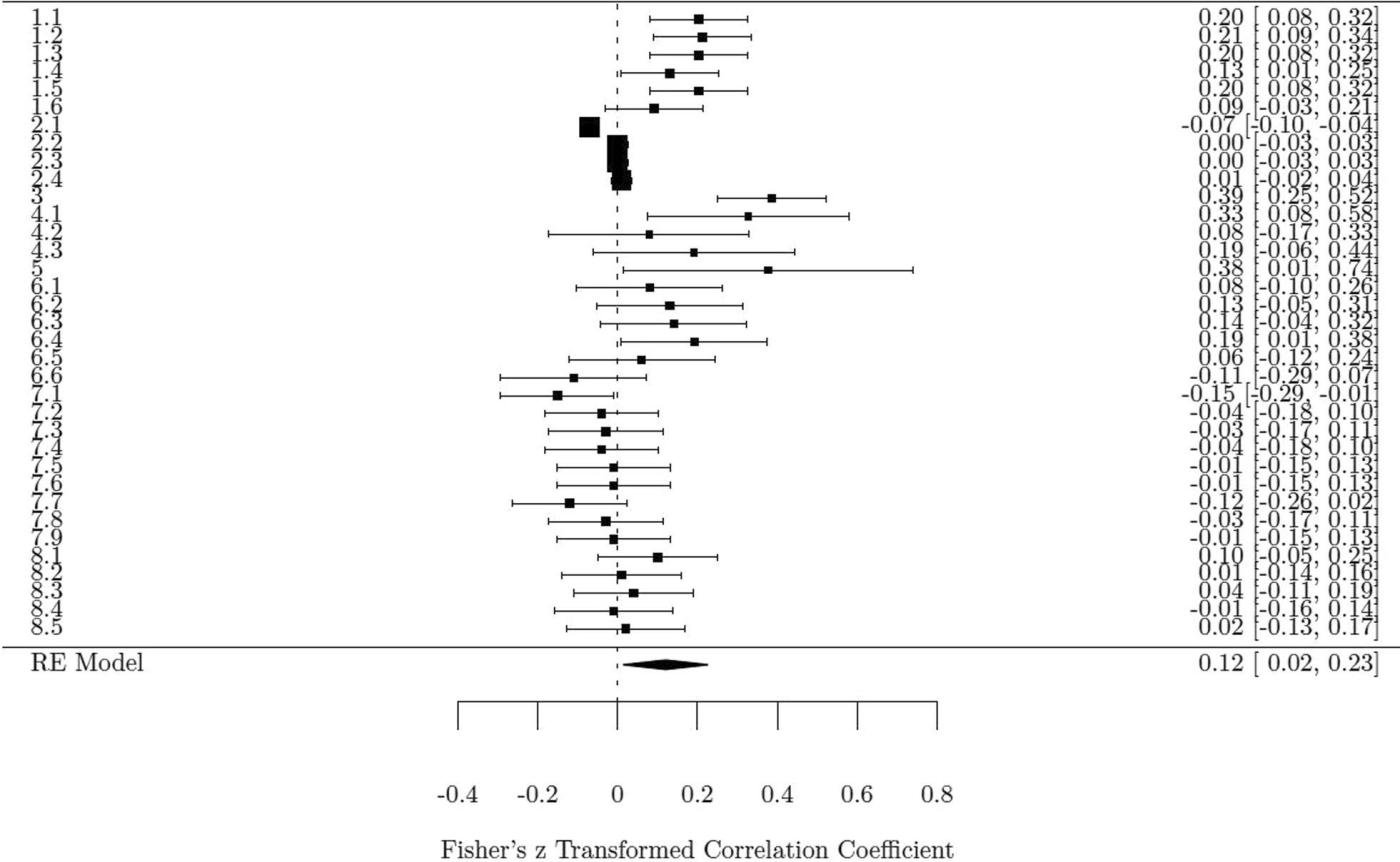
The forest plot of effect sizes. *Notes* Each square represents the effect size of the study result with 95% confidence interval. The size of the symbol is proportional to the sample size of the study. On the left, in the first digit, the study number is presented (1–8), and reported results are enumerated if more than one result is available

As studies used multiple indexes for EFs, for most studies there were multiple effect sizes. Consequently, the actual number of effect sizes used in the analysis was 35 nested within 8 studies. Pearson correlation coefficients were transformed into the effect size measure of Fisher’s *z*. The total effect size was estimated with multi-level meta-analysis. The multivariate meta-analysis model showed a small but significant mean effect size, *z* = 0.12, 95% *CI* (0.02–0.23), *p* = 0.02, indicating that baseline EF was positively associated with later PA behavior. Forest plot for the effect sizes for each study and the total effect size are presented in Fig. 2. The test of heterogeneity was significant, *χ2* (34) = 145.29, *p* < 0.001, *I*^2^ = 87.45%, revealing a high variance across studies.

We further tested for a set of moderators—type of EF measurement (direct/indirect), EF component (updating/inhibition/shifting),^1^ age category of the sample (children/young adults/older adults), and the time frame of the study (one week/twelve months/six years). For the type of EF measurement, out of 35 individual results, 7 of them were indirect, and 25 direct. For the EF component, 25 individual results were available, 6 were coded as updating, 12 as inhibition, and 7 as shifting. For the age category, 1 individual result was excluded since the sample was mixed aged, other than that, 4 were children, 25 were young adults, and 5 were older adults. Lastly, for the time frame of the study, out of 35, 4 were six years, 5 were twelve months, and the rest 26 were one week. The tests of the moderators yielded that the effect of the type of EF measurement (*QM*(1) = 0.974, *p* = 0.32), EF component (*QM*(2) = 0.034, *p* = 0.98), age category (*QM*(2) = 1.239, *p* = 0.54), and time frame of the study (*QM*(2) = 1.64, *p* = 0.44) were not significant. Residual heterogeneity was significant at each, suggesting that the moderator could explain only a small portion of the variance across studies.

**Fig. 3:**
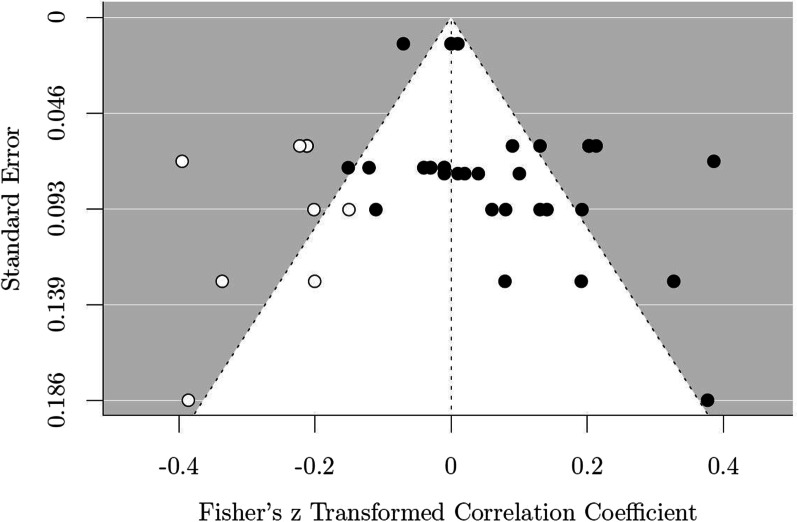
The funnel plot. *Notes* Each black dot represents one study result that was included in the meta-analysis. White dots represent the effect size of hypothetical unpublished results

When publication bias was assessed, the funnel plot with trim and fill added eight hypothetical missing studies (see Fig. 3). Including these studies decreased the total effect size and it was no longer significant, *z* =  − 0.0002, 95% *CI* (− 0.05–0.05), *p* = 0.995.

## Discussion

The present meta-analysis was conducted to examine the relationship between EFs and PA behavior with a specific focus on the direction of the relationship. In the literature, there has been a large interest in exploring the beneficial effects of acute or regular PA on EFs [48–51]. However, the role of EFs as part of self-regulatory processes, and its effect on PA behavior was examined scarcely, in particular from a meta-analytic perspective (for an exception see Gray-Burrows et al.[32]). For this purpose, systematic searches have been carried out, and prospective studies where the direct relationship between baseline EF and later PA behavior was assessed in healthy populations of any age were selected. Eight studies were found eligible that had good to fair quality. The results of the meta-analysis showed that the total effect size for the relationship between EF and PA behavior was small but significant. In short, when results across studies were synthesized quantitatively, it was revealed that baseline EFs predicted later PA behavior significantly. Our results supported the point of view that EFs should be considered as one of the potential determinants of PA behavior. The effect size of our meta-analyses was comparable with the results of Gray-Burrows et al. [32] with regard to physical activity, despite differences in the studies included (i.e. we included prospective studies only, while Gray-Burrows et al. also included cross-sectional studies).

High heterogeneity was observed across studies that might have resulted from different study design characteristics, varying time intervals between t_1_ (when assessing EFs) and t_2_ (when assessing PA), age groups of the study samples, and the heterogeneous measurement methods used for EFs and PA behavior. Some studies used self-report measures while others used performance measures. Especially, large variance of EF measures employed across studies created a challenge to explore further EFs and PA relationship differentiating for EF component (inhibition, updating, shifting) as each of these components play a differentiated role in the self-regulation of PA [11]. It was considered that it was not completely clear which component was measured with the specific tasks, and that some tasks might tap on more than one EF component while other tasks even tap on all components. In addition, tasks differ in the way how much they tap on particular EFs and also how much they tap on cognitive domains beyond EFs (the task-impurity problem, [16]). There is a necessity for the application and development of domain specific EF tests, and standardization of EFs’ measurement in order to be able to compare results across studies and to differentiate for EF domains.

### Limitations

It should be pointed out that the number of studies analyzed was limited which constituted a weakness of the present meta-analysis. As relatively few studies investigated the impact of EFs on PA behavior so far, it was possible to include only eight studies. Due to the small sample size, the precision of the parameter estimates regarding confidence intervals might be rather low. Another limitation of the present study was although the direction of the EF-PA relationship was established with timeline (i.e., EFs were assessed before PA), it was not possible to draw causal inferences with full confidence due to the lack of experimental manipulation studies. As the number of included studies and effects was quite low, the presented moderation analyses should also be interpreted with caution as the number of included studies and effects at each level of the moderator was even lower. Furthermore, the quality of the included studies was fair to good but also showed some uncertainty with regard to quality (see Table 2). Lastly, the main focus of the present meta-analysis was solely EFs and its subcomponents based on a validated theoretical model (i.e., the unity/diversity model of Miyake et al. [17]). However, other cognitive processes such as problem solving, abstract reasoning, and prospective memory might be related to adherence to PA behavior when applying different theories of EFs as well as processes beyond EFs.

### Future directions

Despite limitations, the present meta-analysis showed that baseline EFs were significantly and positively associated with later PA behavior. For future research, it is highly encouraged to take into account the potential bidirectional relationship between EFs and PA behavior [52] and notably to clarify the role of EFs for the self-regulation of PA, differentiating for components of EFs. Large-scale longitudinal studies and RCTs of high quality are needed to reach this goal and to better document causal links. Furthermore, some of the studies included in the present meta-analysis (1, 4, 6, 7) provided evidence for the moderator role of EFs on the intention-PA behavior relationship. It is reasonable to argue that EFs and self-regulatory processes are only relevant when there is a PA goal and thus intention, which might also explain the reported significant but small overall effect size for EFs and PA relationship. Therefore, we suggest that the effect of EFs as a moderator of the intention-behavior relationship might be more relevant than the direct effect of EFs on PA behavior [23, 26]. Future studies should address the moderator role of EFs in relation to PA behavior.

In addition, the mechanisms by which EFs support PA behavior are not fully understood. For example, Kelly and Updegraff [53] documented that activity substitution positively mediated the relationship between cognitive flexibility (i.e., shifting) and PA, where participants recorded if they engaged in the planned PA, an alternate activity for substitution, or none. People with higher shifting abilities showed more substitution behavior and therefore higher PA levels. As in this study, mediator variables of the EFs-PA relationship should be explored and further investigated to learn more about these mechanisms. Furthermore, the potential roles of other cognitive processes such as problem solving besides EFs should be explored in the prediction of PA behavior.

### Practical implications

These findings can be translated into effective intervention strategies to improve PA behavior, taking into consideration individual differences in EF components. For PA promotion programs it could be suggested to target improvement of EFs (e.g., computer based training of EFs or exercise) as well as PA intentions, and to implement efficient self-regulatory strategies by which EF component deficits could be compensated for. For example, Pfeffer and Strobach [47] reported that for people with lower to average updating performance, planning significantly predicted PA behavior. It was shown that planning could compensate for poor updating abilities especially when intentions are high. Evidence-based PA intervention programs are highly encouraged, targeting to improve EFs and efficient strategies should be developed to compensate for lower performances of EF components in order to overcome barriers to initiate and maintain PA.

## Conclusions

Our meta-analysis showed that EFs might be a relevant predictor of PA behavior. As the number of included studies and effects was low and the heterogeneity was high, further research is needed to examine the effect of EFs on PA and the moderating role for the intention-behavior gap. Based on the GRADE instrument by considering number of included studies, study designs, risk of bias, heterogeneity, differences in study population, publication bias, and differences in outcome measures, we assess the quality of evidence of our meta-analysis as low-to-moderate as we are confident that the true effect is likely to be close to the estimate of the effect, but there is a possibility that it is substantially different. In particular, randomized controlled trials of high quality manipulating EFs and assessing transfer effects on PA are needed to examine causal relationships.

## Supplementary Information

**Additional file 1**. Detailed information on study characteristics included in the meta-analysis.

## Data Availability

All data generated or analyzed during this study are included in this article as supplementary material.

## Footnotes

1 To perform the moderation analysis on the EF components (updating/ inhibition/ shifting), from the total of 35 tests, we could identify 6 tests mainly tapping on the updating component (e.g., the visual memory task, the n-back task), 12 tests mainly tapping on the inhibition component (e.g., Stroop task, Flanker task, go/ no-go task), as well as 7 tests mainly tapping on the shifting component (e.g., the task cueing paradigm, the alternating runs paradigm). The remaining 10 tests were not tapping on individual EF components (e.g., self-motivation) or not tapping on the central three EF components (e.g., dual tasking).
