# Supplementary material for: Do executive functions predict physical activity behavior? A meta-analysis
Source: BMC Psychol. 2023 Feb 2;11:33. doi: 10.1186/s40359-023-01067-9 (PMC9893561; doi:10.1186/s40359-023-01067-9)
Supplement: Supplementary file 1 — Additional file 1. Detailed information on study characteristics included in the meta-analysis. [file 40359_2023_1067_MOESM1_ESM.pdf]

| STUDY | TYPE OF STUDY | PA measure | PHYSICAL ACTIVITY | EF measure | EF component | EXECUTIVE FUNCTION | Expected direction | Correlation r(adjusted) | P | N | AGE GROUP | Time frame |
|---|---|---|---|---|---|---|---|---|---|---|---|---|
| 1 | observational | subjective | self-reported PA | subjective | | executive function deficits total | negative | 0,2 | < .001 | 260 | young adults | 1 week |
| 1 | observational | subjective | self-reported PA | subjective | | self-management of time dificits | negative | 0,21 | < .001 | 260 | young adults | 1 week |
| 1 | observational | subjective | self-reported PA | subjective | | self-organization deficits | negative | 0,2 | < .001 | 260 | young adults | 1 week |
| 1 | observational | subjective | self-reported PA | subjective | | self-restraint deficits | negative | 0,13 | < .05 | 260 | young adults | 1 week |
| 1 | observational | subjective | self-reported PA | subjective | | self-motivation deficits | negative | 0,2 | < .001 | 260 | young adults | 1 week |
| 1 | observational | subjective | self-reported PA | subjective | | emotion regulation deficits | negative | 0,09 | > .05 | 260 | young adults | 1 week |
| 2 | observational | objective | average daily MVPA | objective | | selective attention | positive | -0,07 | < .001 | 6069 | children | 6 years |
| 2 | observational | objective | average daily MVPA | objective | | attentional control | positive | 0 | > .05 | 6069 | children | 6 years |
| 2 | observational | objective | average daily MVPA | objective | Updating | working memory | positive | 0 | > .05 | 6069 | children | 6 years |
| 2 | observational | objective | average daily MVPA | objective | Inhibition | response inhibition | positive | 0,01 | > .05 | 6069 | children | 6 years |
| 3 | obvervational | objective | average daily PA | objective | Inhibition | inhibition | positive | 0,368 | <.01 | 208 | adults | 1 week |
| 4 | observational | subjective | vigorous PA | objective | Inhibition | response inhibition reaction time | negative | 0,316 | < .05 | 64 | young adults | 1 week |
| 4 | observational | subjective | vigorous PA | objective | Inhibition | go trials reaction time | negative | 0,079 | > .05 | 64 | young adults | 1 week |
| 4 | observational | subjective | vigorous PA | objective | Inhibition | nogo trials reaction time | negative | 0,189 | > .05 | 64 | young adults | 1 week |
| 5 | observational | objective | light PA | subjective | Shifting | task switching perceptions | positive | 0,36 | 0,07 | 32 | young adults | 1 week |
| 6 | observational | subjective | vigorous PA | objective | Inhibition | go/no-go inhibition | negative | 0,08 | > .05 | 118 | young adults | 1 week |
| 6 | observational | subjective | vigorous PA | objective | Inhibition | stop-signal inhibition | negative | 0,13 | > .05 | 118 | young adults | 1 week |
| 6 | observational | subjective | vigorous PA | objective | Updating | visual memory updating | positive | 0,14 | > .05 | 118 | young adults | 1 week |
| 6 | observational | subjective | vigorous PA | objective | Updating | n-back updating | positive | 0,19 | < .05 | 118 | young adults | 1 week |
| 6 | observational | subjective | vigorous PA | objective | Shifting | task-cueing shifting | negative | 0,06 | > .05 | 118 | young adults | 1 week |
| 6 | observational | subjective | vigorous PA | objective | Shifting | alternating runs shifting | negative | -0,11 | > .05 | 118 | young adults | 1 week |
| 7 | RCT | subjective | vigorous PA | objective | Inhibition | go/no-go inhibition | negative | -0,15 | < .05 | 191 | young adults | 1 week |
| 7 | RCT | subjective | vigorous PA | objective | Inhibition | stop-signal inhibition | negative | -0,04 | > .05 | 191 | young adults | 1 week |
| 7 | RCT | subjective | vigorous PA | objective | Updating | visual memory updating | positive | -0,03 | > .05 | 191 | young adults | 1 week |
| 7 | RCT | subjective | vigorous PA | objective | Updating | n-back updating | positive | -0,04 | > .05 | 191 | young adults | 1 week |
| 7 | RCT | subjective | vigorous PA | objective | Shifting | task-cueing shifting | negative | -0,01 | > .05 | 191 | young adults | 1 week |
| 7 | RCT | subjective | vigorous PA | objective | Shifting | alternating runs shifting | negative | -0,01 | > .05 | 191 | young adults | 1 week |
| 7 | RCT | subjective | vigorous PA | objective | Inhibition | inhibition factor score | negative | -0,12 | > .05 | 191 | young adults | 1 week |
| 7 | RCT | subjective | vigorous PA | objective | Updating | updating factor score | positive | -0,03 | > .05 | 191 | young adults | 1 week |
| 7 | RCT | subjective | vigorous PA | objective | Shifting | shifting factor score | negative | -0,01 | > .05 | 191 | young adults | 1 week |
| 8 | intervention | objective | class attendance | objective | | dual task % error | negative | 0,1 | > 0.5 | 177 | older adults | 12 months |
| 8 | intervention | objective | class attendance | objective | Inhibition | stroop task cost | negative | 0,01 | > 0.5 | 177 | older adults | 12 months |
| 8 | intervention | objective | class attendance | objective | Inhibition | flanker proportional cost | negative | 0,04 | > 0.5 | 177 | older adults | 12 months |
| 8 | intervention | objective | class attendance | objective | | WCST % per errors | negative | -0,01 | > 0.5 | 177 | older adults | 12 months |
| 8 | intervention | objective | class attendance | objective | Shifting | task-switching costs | negative | 0,02 | > 0.5 | 177 | older adults | 12 months |

Notes. RCT = Randomized controlled trial, PA = Physical Activity, MVPA = Moderate/Vigorous Physical Activity, EF = Executive function, N = Sample size
